# Efficacy and Predictability of Cyclin-Dependent Kinase 4/6 Inhibitors in HER2-Positive Breast Cancer

**DOI:** 10.3390/cancers17172788

**Published:** 2025-08-26

**Authors:** Muhammad Shahmir Abbasi, Muhammad Zubair Afzal, Tayyaba Sarwar, Holly A. Gamlen-Steves

**Affiliations:** 1 Department of Geriatric Medicine, Rush University Medical Center, Chicago, IL 60612, USA; 2 Comprehensive Breast Program, Department of Medical Oncology, Dartmouth Cancer Center, Lebanon, NH 03755, USA; muhammad.z.afzal@hitchcock.org; 3 Department of Internal Medicine, Dartmouth Hitchcock Medical Center, Lebanon, NH 03755, USA; tayyaba.sarwar@hitchcock.org (T.S.); holly.a.gamlen@hitchcock.org (H.A.G.-S.)

**Keywords:** cyclin-dependent kinase 4/6 inhibitor, HER2 positive, targeted therapy, breast cancer

## Abstract

HER2-positive breast cancer represents a biologically aggressive subtype associate with poor prognosis, despite advances in targeted therapies. Cyclin-dependent kinase 4/6 inhibitors (CDK4/6i), initially approved for hormone-receptor-positive, HER2-negative disease, are now being explored in HER2-positive settings due to their mechanistic synergy with the HER2 signaling pathway. This review synthesizes evolving clinical evidence from trials and highlights further research into biomarker discovery. CDK4/6i may redefine treatment paradigms in HER2-positive breast cancer, offering a potential, non-chemotherapy option with durable benefit in select patient populations.

## Simple Summary

Despite the advent of targeted therapy for HER2-positive breast cancer with specific inhibitors, it still remains a challenge to treat. Many patients respond well at first, but resistance to treatment can develop, leading to cancer progression. Cyclin-Dependent Kinase 4/6 inhibitors are a newer class of cancer medications that can slow cancer growth by blocking proteins needed for cells to divide and are showing potential of being an additional therapeutic option for this cancer subtype. This review explains how these medications work, what clinical trials are teaching us about their benefits, and how future use could be guided by personalized biomarkers.

## 1. Introduction

Human Epidermal Growth Factor Receptor 2-positive (HER2+) breast cancer (BC) accounts for 15–30% of all BC subtypes and is recognized as a more aggressive pathology compared to Hormone Receptor-positive (HR+)/HER2-negative (HER2−) BC, with HER2+ BC patients having a poorer prognosis [1,2]. HER2 is a subtype of the Receptor Tyrosine Kinase (RTK) family, which consists of four transmembrane proteins that act through a similar secondary messenger system and are encoded by the Erythroblastic Oncogene B (ERBB): HER1/ERBB1 (also known as EGFR), HER2/ERBB2, HER3/ERBB3, and HER4/ERBB4 [3]. These receptors have an extracellular binding domain and an intracellular tyrosine kinase domain, which are typically activated by homodimerization or heterodimerization, usually following ligand binding. They are involved in cell growth and proliferation [4]. The significance of HER2 receptors in cancerous cells was initially recognized in rat models, and HER2 overexpression was soon identified as an indicator of aggressive breast cancer in humans [5,6]. As HER2 does not require a ligand to bind to it, the current hypothesis is that overexpression or amplification of HER2/ERBB2 allows it to homodimerize with itself or heterodimerize with other HER subtypes. This leads to the overactivation of cellular propagation and evasion of apoptosis through the RACS-MAPK and PI3K-AKT pathways, promoting cell cycle continuity and resulting in tumorigenesis [4,7]. The American Society of Clinical Oncology (ASCO) and the College of American Pathologists have provided guidelines on testing and diagnosing HER2+ breast cancer (BC), most recently updated in 2023 [8]. The guidelines also note the newly reported subcategory of HER2-low, as defined in the DESTINY-04 trial, which includes IHC 1+ or 2+/ISH non-amplified cases that would typically be considered HER2-negative but are distinct from IHC 0 cases based on their response to antibody-drug conjugate (ADC) therapy in the trial [9]. However, it was acknowledged that data on its prognostic value are still insufficient. Further retrospective reviews assessing the validity of this newer phenotypic category have yielded mixed results [10,11,12]. Patients with Estrogen/Progesterone-Receptor-positive and HER2+ are known as “Triple Positive” BC, a different phenotypic entity from Hormone-Receptor-Negative HER2+ BC, with the latter having worse disease-free survival, overall survival and higher risk of recurrence.

As treatment options in HER2+ breast cancer continue to expand, the role of biomarkers has become increasingly important to personalize therapy and guide clinical decision-making. Biomarkers can help identify patients most likely to respond to specific targeted treatments, monitor efficacy, and detect mechanism of resistance. In the context of CDK4/6i, both intrinsic tumor features and acquired molecular alterations influence clinical outcomes, making biomarker development and validation a critical component of ongoing research [13,14,15].

Beyond prognostication, HER2+ status enables patients to receive a range of targeted therapies. Trastuzumab was the first humanized monoclonal antibody to demonstrate reduced mortality and increased overall survival in HER2+ breast cancer patients [16,17,18]. The exact mechanism of trastuzumab’s antineoplastic activity is not yet fully defined. Some studies suggest that it blocks the extracellular domains of HER2 receptors, preventing dimerization and tyrosine kinase activation, while others hypothesize a role for antibody-mediated cytotoxicity, as well as the inhibition of angiogenesis [4,19]. Recent clinical trials have led to the approval of newer antibody therapies, including pertuzumab in combination with trastuzumab and margetuximab. Other HER2-targeted therapies include small-molecule tyrosine kinase inhibitors (TKIs), such as lapatinib, neratinib, or tucatinib, as well as antibody-drug conjugates (ADCs) like trastuzumab emtansine or trastuzumab deruxtecan [8].

Current therapeutic strategies involve combining HER2-targeted therapies with endocrine therapies (if HR+) and chemotherapy. Cyclin-dependent kinase inhibitors (CDKIs) are an additional class of medications that have been extensively tested in HR+HER2− BC and are recommended in current guidelines. Ongoing research regarding CDKIs in HER2+ patients has highlighted their unique role in preventing resistance to HER2-targeted therapies. This review will discuss the current trial evidence for the use of CDKIs in HER2+ BC.

## 2. Use of Cyclin-Dependent Kinase Inhibitors as Anticancer Medications

In mammalian cells, cell division is divided into a four-stage process with checkpoints to ensure complete fidelity and prevent DNA aberrations from being passed along the cell line. These stages include two “gap” phases (G1 and G2), the “S phase” for DNA synthesis, and the “M phase” for mitosis. The decision for a cell to initiate division by entering G1 versus remaining quiescent (G0) depends on extracellular stimuli, which encourage the cell to progress beyond the initial restriction point in late G1. Once this point is passed, the cell can proceed to DNA synthesis without further stimulus [20]. After DNA replication, the cell enters G2, which prevents progression to mitosis if DNA repair is incomplete [21,22]. These cell-cycle checkpoints depend on various interactions, including Cyclin-Dependent Kinase (CDK) complexes and inhibition by Cyclin-Dependent Kinase Inhibitors (CKIs). CKIs are further divided into INK4 proteins, which prevent CDK4/6 from binding to Cyclin D, including p16INK4a, p15INK4b, p18INK4c and p19INK4d, and CIP/KIP proteins that inhibit the CDK-Cyclin D complexes comprising p21Cip1, p27Kip1, and p57Kip2. The G1 phase is regulated by interactions between CDK4/6, Cyclin D, Retinoblastoma protein (Rb) and E2F transcription factor. D-type Cyclins (D1, D2 or C3) combine with either CDK4 or CDK6, activating them, which, in turn, inactivate Rb by phosphorylation, releasing E2F, which subsequently activates genes required to progress through the S phase [22,23].

Altered activity of CDKs has been noted in a variety of cancer types. CDK1 complexes with Cyclin B act as a gatekeeper between late G2 and M phase by preventing premature entry into mitosis, which allows errors in DNA replication to be identified, along with driving key mitotic events including chromosome condensation and nuclear envelope breakdown. Inactivation of CDK1-Cyclin B during anaphase allows the cell to exit mitosis and complete cytokinesis. Overexpression of CDK1 and Cyclin B has been observed in melanoma, uterine sarcomas, and colorectal cancers, which likely allows chromosomally unstable cells to proceed through mitosis, thereby enabling tumorigenesis [22,24]. Aberrations in the Cyclin D-CDK4/6-RB pathway have been observed in breast cancer cells. Up to 50% overexpression of Cyclin D1 has been noted in mammary cancer cells, some of which is due to amplification of the CCND1 gene, seen in up to 20% of breast cancers [25,26]. This is especially prominent in luminal A- and B-type tumors, likely due to a positive feedback loop with estrogen receptors. It is also observed in HER2+ breast cancer, because of increased signaling from ERBB2. Mutations in CKIs have also been implicated, with reduced expression of p16INK4A and p27Kip1 seen less frequently in luminal A type breast cancers compared to others. Triple-negative breast cancer (TNBC) has the greatest frequency of Rb inactivity, whereas the luminal type usually has intact Rb functionality [26,27,28,29].

The first CDKI introduced was flavopiridol, considered a first-in-class drug, which had non-selective inhibition against CDK1, CDK2, CDK4/6, and CDK7. It was tested against a broad range of cancers in clinical trials. However, due to a high incidence of toxicities coupled with limited therapeutic response, no further trials were conducted beyond phase II [22,30]. Next came roscovitine, which similarly had non-selective inhibition of CDKs, except for CDK4/6. However, it failed to demonstrate clinical significance in early trials. Subsequent generations moved away from pan-CDK inhibition and focused on multi-target CDK selectivity, offering improved potency and reduced toxicity compared to their predecessors. Several drugs, including dinaciclib, AT7519, R547, SNS-032, AZD5438, and AG-024322, were developed. As of now, none have been approved for clinical use, as they have been unable to demonstrate efficacy without significant toxicities. Dinaciclib has been the most extensively explored, with a phase III trial comparing its efficacy against ofatumumab for relapsed/refractory chronic lymphocytic leukemia. However, the trial was terminated prior to obtaining a sufficient sample size (though this was unrelated to safety or efficacy issues), preventing a full conclusion from being drawn [22,31,32,33,34].

The paradigm shifted with the introduction of highly selective CDK4/6 inhibitors, the first of which was PD-0332991, later known as palbociclib. Its antineoplastic activity was quickly identified because it induced G1 arrest, which either prevented tumor growth or even led to tumor regression, as observed in preclinical studies involving murine models, human ex vivo cancer cells, and xenografts [35,36,37,38,39]. Finn et al. conducted their seminal study using PD 0332991, later known as palbociclib, and assessed its activity against 47 in vitro breast cancer cell lines. A multidrug effect analysis was conducted, which included two important sub-groups: ER-positive cell lines, which were treated with PD 0332991 alone, 4-hydroxytamoxifen only or in combination, and a trastuzumab group, which comprised trastuzumab only, PD 0332991 only or in combination. ER+ cells were most sensitive, and 10/16 HER2-amplified cell lines showed sensitivity. PD 0332991 inhibited the growth of luminal ER-positive and HER2+ cancer cells, showed synergistic activity with antiestrogen therapy and trastuzumab, and reversed resistance to tamoxifen [40]. However, it also demonstrated reduced activity in basal-type cancer cells, likely due to decreased expression of Rb [26,27,28,40]. These findings became the basis for randomized clinical trials investigating the efficacy of CDK4/6 inhibitors in HR+/HER2− breast cancers.

## 3. CDK4/6 Inhibitors in Hormone-Receptor-Positive HER2-Negative Breast Cancers

To fully recognize the role of CDK4/6i in HER2-positive breast cancer, an understanding of their role in HR+/HER2− breast cancer treatment is needed. The exploratory phase Ib/II PALOMA-1 trial and the confirmatory phase III PALOMA-2 trial demonstrated a significant difference in progression-free survival (PFS) in HR+/HER2– advanced breast cancer (ABC) patients receiving palbociclib plus letrozole versus letrozole alone, which resulted in accelerated approval of the combination therapy by the FDA [41,42,43,44]. Ribociclib and abemaciclib followed a similar path, based on the results from the MONALEESA (2, 3, and 7) and MONARCH (1, 2, and 3) trials, respectively [45,46,47,48,49,50,51]. Additionally, they demonstrated a statistically significant improvement in overall survival (OS), unlike palbociclib. However, subsequent comparative analyses and real-world data have yet to confirm this difference [52,53,54].

Dalpiciclib is another CDK4/6 inhibitor (CDK4/6i) approved in China as part of a combination regimen for ABC. It demonstrated improved PFS when combined with fulvestrant (15.7 months) versus fulvestrant plus placebo (7.2 months) in patients who relapsed on previous endocrine therapy (ET) in the DAWNA-1 trial. It also showed an increase in median PFS in hormone-sensitive patients when combined with aromatase inhibitors in the DAWNA-2 trial [55,56].

Abemaciclib was subsequently approved by the FDA for use as adjuvant therapy when combined ET, in early, high-risk breast cancer, as demonstrated in the monarchE trial. In a population of 5637 patients, there was a significant increase in invasive disease-free survival (iDFS) with abemaciclib plus ET administered for two years compared to standard ET (92.2% vs. 88.7%), with a sustained benefit noted four years after completion of therapy according to an interim analysis [57,58,59]. Ribociclib was next to be approved by the FDA as a combination with letrozole for adjuvant therapy, based on the NATALEE trial, which showed a three-year iDFS rate of 90.7% when used in combination with ET, compared to 87.6% with ET alone. However, palbociclib failed to demonstrate such benefits in the adjuvant setting, in either the PALLAS or PENELOPE-B trials [60,61,62,63].

In the neoadjuvant setting, the neoPAL and CORALEEN trials compared CDK4/6i plus ET combinations with chemotherapy, with neither demonstrating a significant benefit over chemotherapy. The PALLET, FELINE, and neoMONARCH trials assessed CDK4/6i plus ET versus ET alone; the combination demonstrated significant antiproliferative activity, with complete cell cycle arrest and reduction in Ki-67 expression. However, none of the CDK4/6i plus ET combinations were able to improve the pathological complete response (pCR), which may be due to the short duration of therapy and the lack of overall survival (OS) data [64,65,66,67,68].

Clinical trials involving HR+/HER2– breast cancer (BC) and CDK4/6i, including those currently ongoing, are listed in Table 1. The newer trials have multiple areas of focus, including identifying relevant biomarkers for patients receiving CDK4/6i, exploring therapeutic options for patients who progress after CDK4/6i therapy, and assessing potential alternatives to the current standard of care—ET plus CDK4/6i. A list of drug categories and mechanisms, as well as a list of abbreviations are provided in Supplemental Materials in Table S1 and Table S2, respectively.

## 4. Significance of CDK4/6 Inhibitors in HER2-Positive Breast Cancer

After the success of CDK4/6i as a therapeutic option for HR+/HER2– breast cancer, the focus naturally shifted toward HER2+ breast cancer.

HER2 interacts with CDK4/6 through multiple intracellular pathways; its overstimulation leads to increased activity of the RAS–MAPK and PI3K–AKT pathways, which subsequently upregulate the expression of cyclin D1. Cyclin D1 then forms a complex with CDK4/6 [3,4,22]. The importance of this relationship was highlighted in preclinical studies, where CDK4/6i caused cytostatic inhibition of HER2+ cells. This effect was amplified in the presence of trastuzumab, resulting in greater G0/G1 cell cycle arrest than either agent alone [40]. Despite the use of targeted anti-HER2 therapies, these cancers often develop resistance through various mechanisms, such as mutations in the HER2 receptor itself or increased activity of downstream signaling pathways. Elevated PI3K–AKT activity may be mediated by loss-of-function mutations in PTEN (in up to 5% of HER2+ breast cancers) or activating mutations in the PIK3CA gene (seen in approximately 20% of HER2+ cases). Even with inhibition of the PI3K–AKT pathway, somatic mutations in the MEK–ERK cascade can provide an escape mechanism, as observed in cancers resistant to anti-HER2 TKIs like tucatinib.

By acting downstream of these signaling pathways, CDK4/6i offers a potential strategy to overcome such resistance [132,133,134,135].

Another mechanism of resistance is crosstalk between Estrogen Receptors (ERs) and HER2. An ER influences the cells primarily through hormone-activated gene expression by binding to its Estrogen Response Elements (EREs) in the promoter region of target genes, known as the classic genomic pathway. By attaching to other transcription factors like Activator Protein-1 Transcription Factor, it can stimulate cell activity through what is called the ERE-independent genomic pathway. Finally, estrogens are noted for having non-genomic actions as well; membrane ER can activate membrane RTKs, most importantly HER2/ERBB2. These pathways provide an acquired resistance by increased ER activity, noted in cancer cells treated with antiHER2 TKIs. Similarly, cancer cells with resistance to ET are noted for having increased HER signaling, with the pathway providing an escape [136,137]. HER2 and subsequent signaling cascades influence ER genetic transcription, in turn reducing the expression of ER, effectively limiting the target sites for ET. This bidirectional stimulation is a possible reason for HR+HER2+ cancers responding differently to treatments which focus either on ET or antiHER2 therapy. Combined receptor blockade was tested in clinical trials, which showed improved PFS, but did not significantly affect OS in advanced breast cancers. There was no significant difference between combined therapy from standard of care in neoadjuvant settings either [15,136,137,138,139,140].

Cyclin D-CDK4/6-Rb is a common pathway for both hormone receptors and RTK. Co-targeting Rb with CDK4/6i in addition to combined ET and antiHER2 therapy demonstrated robust results in preclinical models, demonstrating triple-targeted therapy as an option to overcome resistance [25,26,138,139,140,141,142].

The role of CDK4/6i and their efficacy in combination with antiHER2 therapy was further demonstrated in the trials discussed below.

## 5. CDK4/6 Inhibitors in Advanced/Metastatic, HER2-Positive Breast Cancer

DAP-Her-01 (NCT04293276) was a single-arm, phase II trial in which 41 enrolled patients with metastatic breast cancer (MBC) received a combined regimen of pyrotinib and dalpiciclib. Only 7% (3/41) of patients had not received prior systemic therapies. The median progression-free survival (PFS) was 11.0 months (95% CI = 7.3–19.3), with an estimated 12-month PFS rate of 44.7%. Ten deaths occurred after disease progression. The OS rates at 12 and 18 months were 90.0% and 82.5%, respectively. One patient discontinued pyrotinib due to developing heart failure. Further analysis revealed that HR-negative patients tended to achieve a higher overall response rate (ORR) (81.8% vs. 55.6%) and longer PFS (19.3 vs. 9.1 months) compared to HR+/HER2+ patients [143]. Subsequently, the study investigators developed the DAP-Her-02 trial (NCT05328440), which aims to compare pyrotinib plus dalpiciclib and fulvestrant in HR+/HER2+ patients versus a combination of pyrotinib, dalpiciclib, and inetetamab in HR−/HER2+ patients [144].

NCT03530696 was a phase II trial assessing the efficacy of combining trastuzumab emtansine (T-DM1) with palbociclib versus T-DM1 alone in a population of 55 patients with MBC. The study was eventually switched to a single-arm design due to low recruitment, evaluating the efficacy of the combination therapy. About two-thirds of patients had received prior therapies for breast cancer, and 86.3% had brain metastases. The median PFS for T-DM1 alone was 8.3 months, compared to 16.9 months for the combination group, with ORRs of 18.2% vs. 42.9%, respectively. The OS for the combination therapy was 35.1 months [145]. Though these are preliminary data, these demonstrate the potential synergistic effect of CDK4/6 inhibitors and anti-HER2 therapy.

In a phase Ib/II trial (NCT03054363), forty-two women with metastatic breast cancer (MBC) and a history of two prior anti-HER2-based therapies were enrolled. They received a combination of tucatinib (300 mg orally twice daily), palbociclib (75 mg daily for 21 days on, followed by 7 days off), and letrozole (2.5 mg orally daily) in the phase II arm. In the phase Ib portion, palbociclib was administered at 125 mg. Based on a previous trial, a median PFS of 9.6 months was set as the threshold to indicate a 50% improvement over historical controls; however, this was not achieved. The median PFS was 8.7 months overall, with 10.1 months in patients with prior brain metastases versus 6.0 months in those without. The most common grade ≥ 3 adverse event was neutropenia, occurring in 64.3% of patients. Though not statistically significant, the results were still considered meaningful by the study authors, particularly as the regimen offered a treatment option for heavily pretreated MBC [146].

In the phase II monarcHER trial (NCT02675231), 237 patients were randomized into three groups to compare abemaciclib plus trastuzumab with fulvestrant (Group A), abemaciclib plus trastuzumab without fulvestrant (Group B), and standard-of-care trastuzumab plus systemic chemotherapy (Group C). Eligible patients had received at least two prior HER2-targeted therapies. The primary endpoint was median PFS, with a significant difference observed between Group A and Group C (8.3 months vs. 5.7 months), whereas no difference was seen between Group B and Group C [147]. The final OS was 31.1, 29.2 and 20.7 months for groups A, B and C, respectively, with the hazard ratio (HR) for OS comparing group A to group C was 0.71 (95% CI, 0.48–1.05; nominal two-sided *p*-value 0.086), and for group B to group C, the HR was 0.83 (95% CI, 0.57–1.23; nominal two-sided *p*-value 0.365). This demonstrated a numerical improvement in OS for a heavily pretreated population. Intrinsic subtyping using PAM50 and Genefu classifiers showed luminal subtypes were associated with longer PFS (8.6 vs. 5.4 months) and OS (31.7 vs. 19.7 months) compared with non-luminal [148]. Abemaciclib was well tolerated; however, there was a notably increased number of patients with thrombocytopenia.

The phase II SOLTI-1303 PATRICIA trial (NCT02448420) evaluated the efficacy of trastuzumab plus palbociclib, with or without letrozole, in a cohort of 71 patients who had received 2–4 prior lines of anti-HER2-based regimens. Patients were divided into subgroups based on ER/HER2 status. Group A (ER−/HER2+) and Group B1 (ER+/HER2+) received trastuzumab plus palbociclib, while Group B2 (ER+/HER2+) received triple therapy with the addition of letrozole. The PFS rate at 6 months was 33.3% in Group A, 42.8% in Group B1, and 46.4% in Group B2. An interim analysis using PAM50 subtyping showed that patients with luminal breast cancer had longer PFS compared to those with non-luminal disease (median PFS: 10.6 vs. 4.2 months; adjusted hazard ratio = 0.40; *p* = 0.003). Median PFS by intrinsic subtype was 10.6 months (95% CI, 4.1–14.8) in luminal B, 8.2 months (95% CI, 2.2–24.1) in luminal A, 3.8 months (95% CI, 2.1–10.9) in HER2-enriched, and 6.0 months (95% CI, 1.7–11.2) in normal-like tumors [149]. Based on these results, enrollment was stopped early, and two additional cohorts of HR+/HER2+ luminal subtype patients were included. Group C1 received palbociclib plus trastuzumab and endocrine therapy (ET), while Group C2 received a physician’s choice of treatment: trastuzumab emtansine (T-DM1), systemic chemotherapy, or ET plus trastuzumab [147,148]. Group C1 had a longer median PFS compared to C2 (9.1 vs. 7.5 months; stratified HR = 0.52 [95% CI, 0.29–0.94]; two-sided *p* = 0.031), with an objective response rate of 18.9% vs. 8.3%, respectively. These findings demonstrated a statistically significant improvement in PFS for patients with luminal A and B intrinsic subtype breast cancer who received CDK4/6i as part of their regimen [150].

In a separate phase II multicenter trial (NCT04334330), fourteen patients with MBC and brain metastases, naïve to whole-brain radiation therapy, were enrolled to assess the intracranial and extracranial efficacy of combining palbociclib, trastuzumab, pyrotinib, and fulvestrant [151]. The most common grade ≥3 adverse event was gastrointestinal toxicity (diarrhea), occurring in up to 40% of patients, though it was not treatment-limiting. The median PFS was 10.6 months, and the median time to CNS progression was 8.5 months. In comparison, based on previous trial results, patients receiving T-DM1 alone had a median PFS of 5–6.1 months. Prior attempts at dual anti-HER2 therapy with chemotherapy achieved similar CNS-PFS outcomes but were associated with significant adverse events, some leading to patient mortality [151].

The PLEASURABLE trial (NCT03772353) evaluated the combination of dalpiciclib with pyrotinib and either letrozole or fulvestrant in 48 patients with ER+/HER2+ advanced breast cancer (ABC). Of these, 31 had received prior trastuzumab treatment, while 17 were treatment-naïve. A 100% disease control rate was observed, with an objective response rate of 81% in the treatment-naïve group and 61% in the pretreated group. The OS and PFS results are currently pending [152]. NCT05969184 is an ongoing phase II clinical trial assessing the effect of quadruple therapy on two-year PFS in patients with ABC. Up to 94 participants will receive palbociclib (125 mg daily from Day 1 to Day 21 of a 28-day cycle), trastuzumab (every three weeks: 8 mg/kg loading dose followed by 6 mg/kg), pertuzumab (every three weeks: 840 mg loading dose followed by 420 mg), and either letrozole or exemestane at the physician’s discretion [153]. Another ongoing trial (NCT06481956) is investigating subsequent treatment with ribociclib and trastuzumab emtansine (T-DM1) in patients with ABC who have previously been treated with trastuzumab [154]. A phase Ib/II trial (NCT03304080) is evaluating the PFS of ABC patients receiving a combination of dual anti-HER2 therapy (trastuzumab and pertuzumab), anastrozole, and palbociclib. This study also aims to identify potential biomarkers, including cyclin E2 expression, phosphorylated retinoblastoma (pRb), and p16 levels [155].

The ongoing phase III PATINA trial (NCT02947685) has enrolled 518 patients with HR+/HER2+ ABC to assess the efficacy of combining palbociclib with anti-HER2 therapy (trastuzumab ± pertuzumab) and endocrine therapy (ET) versus anti-HER2 therapy and ET alone, following six to eight cycles of standard chemotherapy and trastuzumab as first-line treatment. The median patient age was 53 years, and the majority was white. Approximately 97% had received dual anti-HER2 therapy. At a median follow-up of 53 months, there was a significant improvement in PFS: the experimental (palbociclib) arm achieved a median PFS of 44.3 months (95% CI: 32.4–60.9) versus 29.1 months (95% CI: 23.3–38.6) in the control arm, with a hazard ratio (HR) of 0.74 (95% CI: 0.58–0.94; one-sided *p* = 0.0074). The experimental arm experienced a higher incidence of adverse events, with grade 3 neutropenia being the most common (63.2% vs. 2.0%), along with stomatitis and diarrhea. No treatment-related deaths were reported. OS data are currently pending [156,157]

## 6. CDK4/6 Inhibitors in Early/Limited-Stage, HER2-Positive Breast Cancer

The NA-PHER2 trial was one of the initial phase II exploratory studies investigating the significance of triple-targeted neoadjuvant therapy in patients with ER+/HER2+ unilateral breast cancer. Participants received anti-HER2 therapy (trastuzumab and pertuzumab), palbociclib for RB1 inhibition, and endocrine therapy (fulvestrant). Of the 30 patients enrolled, 29 experienced an objective response prior to surgery, and 8 achieved pCR. A significant mean decline in Ki-67 expression was observed at two weeks, a finding that encouraged further exploration of CDK4/6 inhibition in HER2+ cancers [158]. The study was subsequently extended to include Cohort B, which received palbociclib and dual anti-HER2 therapy without fulvestrant, and Cohort C, which included patients with high Ki-67 and “HER2-low” expression (IHC 1+ or 2+ without gene amplification). Both cohorts showed a decline in Ki-67 expression following therapy [159]. Biomolecular analyses revealed that patients who achieved pCR had tumors characterized by greater immune infiltration and lower ER expression. Additionally, tumors with sustained low Ki-67 expression post-therapy were found to have a luminal A phenotype [160].

The TOUCH trial (NCT03644186) evaluated the efficacy of an adjuvant non-chemotherapy treatment strategy in older patients. Seventy-four participants in the experimental arm received palbociclib, letrozole, and combined anti-HER2 therapy (trastuzumab and pertuzumab), while seventy-three participants in the control arm received paclitaxel and anti-HER2 therapy. Approximately 70% of participants were over 65 years of age. The objective complete response rate was comparable between arms, with 52.8% in both the experimental and control groups. A greater proportion of patients in the palbociclib group underwent breast-conserving surgery (81.9% vs. 67.1%). However, there was a higher incidence of grade III adverse events in the palbociclib group, primarily neutropenia. Despite this, the study demonstrated the viability of a non-chemotherapy option for breast cancer management and highlighted RBsig as a potential predictive biomarker [161,162,163].

The neoPEHP trial (NCT04858516) is looking into neoadjuvant treatment of unilateral, invasive ER+/HER2+ BC with trastuzumab (8 mg/kg loading dose followed by 6 mg/kg) and oral pyrotinib (400 mg po QD) for six cycles plus oral palbociclib (125 mg once a day for 21 days in a 4-week cycle) and oral exemestane. Ref. [164] Similarly, NCT05800756 is an ongoing trial in which patients with early or locally advanced HER2+ BC will receive two cycles of dalpiciclib, dual antiHER2 therapy (trastuzumab and pyrotinib) and letrozole. After the initial cycles, patients will be reimaged to assess for complete or partial response. Those who fail to demonstrate a partial response will switch to dual antiHER2 and chemotherapy, while the other cohort will continue with the remaining cycles of the planned therapy [165]. The trials discussed above are further listed in Table 2 below.

## 7. Predictive and Prognostic Biomarkers of CDK4/6 Inhibitor Responsiveness

Several intrinsic and acquired mechanisms have been associated with therapeutic efficacy. The presence of the functional Rb protein remains a prerequisite for response, as Rb is the direct downstream target of CDK4/6 activity. Conversely, RB1 loss or mutation is a well-established mechanism of resistance [166]. Overexpression of Cyclin E1 or E2, which activates CDK2 and bypasses CDK4/6-mediated cell cycle control, is another mechanism of resistance and has been associated with poor outcomes [15]. p16INK4a can serve as an indirect marker of Rb loss when overexpressed, though its predictive utility remains inconsistent [167,168].

Molecular subtyping through PAM50 profiling has shown that the luminal A and B subtypes derive greater benefit from CDK4/6i, while HER2-enriched and basal-like tumors show attenuated responses. Gene expression signatures like RBsig, developed to assess Rb functional status, and dynamic pharmacodynamic markers like Ki-67suppression post treatment have been used in clinical trials like TOUCH and NA-PHER2 to monitor treatment efficacy. Moreover, circulating tumor DNA (ctDNA), circulating tumor cells (CTCs) and serum thymidine kinase-1 activity are under investigation as liquid biopsy-based biomarkers, offering minimally invasive options for real-time monitoring.

HER2 expression itself is a predictive biomarker, with HER2-low being recently defined as a breast cancer subtype previously classified as HER2-negative. These tumors are now recognized as phenotypically distinct and may benefit from targeted therapies, as suggested by promising results in the DESTINY-Breast04 trial [9]. Retrospective analyses of CDK4/6i in breast cancers reclassified as HER2-low have yielded mixed outcomes [169,170,171]. Ongoing trials are comparing the efficacy of trastuzumab deruxtecan versus endocrine therapy plus CDK4/6i in the ER+/HER2-low subset (NCT06680596, NCT06486883, NCT06585969) [172,173,174].

Emerging genomic and transcriptomic approaches have identified additional alterations—such as FAT1 loss, FGFR pathway activation and MDM2 amplification— that may contribute to intrinsic or acquired resistance [168,175].

## 8. CDK4/6 Inhibitors in Other Types of Breast Cancers

Triple-negative breast cancers (TNBCs) often exhibit mutated or inactivated Rb protein, leading to resistance to CDK4/6i. The basal subtype typically shows resistance, while the luminal androgen receptor (LAR) subtype demonstrates sensitivity [26,176]. It is hypothesized that LAR tumors enter a quiescent state following mitosis and require CDK4/6 activity to re-enter the cell cycle. The ABBICAR trial (NCT06365788) is currently investigating this mechanism by combining abemaciclib with bicalutamide in metastatic TNBC [177]. Similarly, the PAveMenT trial (NCT04360941) is evaluating the combination of avelumab (a PD-L1–targeting monoclonal antibody) and palbociclib in androgen receptor-positive TNBC, with the objective response rate as the primary endpoint [178].

Beyond direct anti-tumor activity, CDK4/6i has also been explored for its ability to reduce chemotherapy-induced myelosuppression and immunosuppression, potentially preserving antitumor immunity. In a phase II trial (NCT02978716), patients with metastatic TNBC received trilaciclib prior to gemcitabine, which resulted in a numerical but not statistically significant improvement in OS: 20.1 months versus 12.6 months for gemcitabine alone [179]. The study also found no significant differences in myelosuppression-related endpoints. This strategy was further examined in the phase III PRESERVE2 trial (NCT04799249), which similarly failed to demonstrate a statistically significant OS benefit with trilaciclib plus gemcitabine compared to the control arm [180,181].

## 9. Future

Despite their known efficacy, most patients with advanced breast cancer develop resistance to CDK4/6i within 12–24 months of initiating therapy. This heterogeneity in response emphasizes the critical need for robust predictive biomarkers to optimize patient selection and therapeutic sequencing. Mechanistically, resistance to CDK4/6i in HER2+BC may arise through an intrinsic versus acquired mutation, or due to activation of alternative signaling pathways such as Aurora kinase A, AKT, and MAPK [182,183]. Current clinical trials are investigating combinations of CDK4/6i with inhibitors targeting these pathways to overcome or prevent resistance and enhance therapeutic efficacy. This is highly valuable as integrating molecular profiling into future studies is essential to develop personalized treatment strategies and optimize patient selection for CDK4/6 inhibitor-based therapies in HER2-positive breast cancer. Trials incorporating real-time ctDNA-based assays and transcriptomic profiling to dynamically assess molecular changes during therapy and preempt resistance development are of the utmost importance.

Another area of unmet need is defining optimal sequencing strategies post-CDK4/6 inhibition. Given the lack of prospective data on effective post-progression therapies, translational studies exploring early resistance mechanisms are needed. The use of multi-omics profiling, coupled with machine-learning-driven biomarker discovery, may facilitate the identification of such markers [175,184].

In parallel, next-generation CDK inhibitors are under development. These include agents highly selective for CDK4 over CDK6 (e.g., PF-07220060, TQB3616), CDK2 inhibitors (e.g., AZD8421, AVZO-021), and novel compounds known as CDK2 PROTAC degraders (e.g., NKT3964), which inhibit CDK2 while preventing the accumulation of cyclin E1 [185,186,187,188,189].

## 10. Conclusions

CDK4/6 inhibitors offer a potential non-chemotherapy treatment option for HER2-positive ABC, particularly for patients with luminal A and B subtypes. The PATINA trial is the first of its kind, demonstrating an improvement in PFS and HR. This trial is well regarded by the oncology community. Although survival data are pending, these are being adopted in clinical practice. Other early phase I and phase II trials are ongoing. Additional phase III trials are needed to validate the data and incorporate the findings into routine clinical practice.

## Figures and Tables

**Table 1:** Clinical trials involving CDK4/6i in HR-positive breast cancer.

Study Name	Intervention	CDK-Inhibitor	Phase	Patient Population	NCT
Efficacy and Safety of CDK4/6 Inhibitors in Combination with Endocrine Therapy in the Neoadjuvant Treatment.	Neoadjuvant therapy: CDK4/6i + Endocrine Therapy (ET)	Abemaciclib; Dalpiciclib; Palbociclib; Ribociclib;	Phase II; single group, open label	Stage II–III breast cancer with history of intolerance or insensitivity to neoadjuvant chemotherapy	NCT06810492 [69]
Endocrine Therapy Plus CDK4/6 in First or Second Line for Hormone (SONIA) Receptor Positive Advanced Breast Cancer	Arm A: Letrozole/Anastrozole + CKD4/6i followed by Fulvestrant Arm B: Letrozole/Anastrozole followed by CDK4/6i + Fulvestrant	Abemaciclib; Palbociclib; Ribociclib	Phase III, randomized, open label	ABC with recurrence or metastasis. Exclusion: prior (neo)adjuvant therapy with AI.	NCT03425838 [70]
Saruparib (AZD5305) Plus Camizestrant Compared with CDK4/6 Inhibitor Plus Endocrine Therapy or Plus Camizestrant in HR-Positive, HER2-Negative (IHC 0, 1+, 2+/ISH Non-amplified), BRCA1, BRCA2, or PALB2m Advanced Breast Cancer (EvoPAR-BR01)	Arm A: Saruparib (AZD5305) + Camizestrant Arm B: CDK4/6i (CDK46/inhibitor) + ET Arm C: CDK4/6i + Camizestrant	Abemaciclib; Palbociclib; Ribociclib	Phase III, multicenter, randomized, open label	Advanced breast cancer (ABC), with germline loss of function BRCA1, BRCA2, or PALB2 mutations	NCT06380751 [71]
Giredestrant Compared with Fulvestrant (Plus a CDK4/6 Inhibitor), in Participants With ER-Positive, HER2-Negative Advanced Breast Cancer Resistant to Adjuvant Endocrine Therapy (pionERA Breast Cancer)	Arm A: Giredestrant + CDK4/6i Arm B: Fulvestrant + CDK4/6i	Abemaciclib; Palbociclib; Ribociclib	Phase II, randomized, open label	ABC, resistance to prior adjuvant ET, ESR1 mutation status. Prior use of adjuvant CDK4/6i is allowed	NCT06065748 [72]
Elacestrant vs. Elacestrant Plus a CDK4/6 Inhibitor in Patients with ER positive/HER2-negative Advanced or Metastatic Breast Cancer	Arm A: Elacestrant monotherapy Arm B: Elacestrant + CDK4/6i	Abemaciclib, Palbociclib, Ribociclib	Phase II, randomized, open label	ABC, ESR1 mutation, must have received at least 2 prior ETs, including a CDK4/6i	NCT06062498 [73]
Implementing Geriatric Assessment for Dose Optimization of Cyclin-dependent Kinase (CDK) 4/6-inhibitors in Older Breast Cancer Patients (IMPORTANT)	Arm A: Frail patient cohort, dose reduced CDK4/6i + ET Arm B: Frail patient cohort, Full initial dose CDK4/6i + ET Arm C: Fit patient cohort, Full initial dose CDK4/6i + ET	Abemaciclib, Palbociclib, Ribociclib	Phase III, multicenter, randomized, open label	ABC, age 70 years and older. Adjuvant treatment with CDK 4/6i is allowed; provided a disease-free interval from treatment end >12 months	NCT06044623 [74]
Levels of Circulating Tumor DNA as a Predictive Marker for Early Switch in Treatment for Patients with Metastatic (Stage IV) Breast Cancer	Step 1: All patients will receive standard of care frontline regimens. Step 2: Arm 1: No modification of therapy. AI/SERD (AI) + CDK4/6i Arm 2: Early switch in therapy. AI/SERD + CDK4/6i to mTOR inhibitor +AI/SERD or PI3K inhibitor + AI/SERD or chemotherapy Step 3: Optional treatment in Arm 1 who experienced progression	Abemaciclib; Palbociclib; Ribociclib	Phase II, randomized, open label, sequential assignment	ABC, no prior systemic therapy including ET unless initiated <30 days from Cycle 1 Day 1. Prior initiation of LHRH agonist or bone-directed agents is allowed	NCT05826964 [75]
Comparing the Immune Modulation Effect of Ribociclib, Palbociclib, and Abemaciclib in ER+/HER2− EBC (ORACLE-RIPA)	Arm A: Palbociclib + Letrozole Arm B: Ribociclib + Letrozole Arm C: Abemaciclib + Letrozole	Abemaciclib; Palbociclib; Ribociclib	Phase II, randomized, open label	Age 20 years or older, stage II–III breast cancer	NCT05766410 [76]
Open-Label Umbrella Study to Evaluate Safety and Efficacy of Elacestrant in Various Combination in Patients with Metastatic Breast Cancer (ELEVATE)	Arm A: Elacestrant + Alpelisib Arm B: Elacestrant + Everolimus Arm C: Elacestrant + Ribociclib/Abemaciclib Arm D: Elacestrant + CDK4/6i Arm E: Elacestrant + Capivasertib	Abemaciclib; Palbociclib; Ribociclib	Phase Ib/II, multicenter, non-randomized, open label	ABC, one or up to two prior ETs in the advanced or metastatic setting, one of which was in combination with a CDK4/6 inhibitor, except for participants in Arm C and D. Arm A exclusion: prior treatment with PI3K inhibitor. Arm B exclusion: prior treatment with mTOR inhibitor	NCT05563220 [77]
PF-07220060 With Letrozole in Adults With HR-positive HER2-negative Breast Cancer Who Have Not Received Anticancer Treatment for Advanced/Metastatic Disease (FourLight-3)	Arm A: PF-07220060 + Letrozole Arm B: CDK4/6i + Letrozole	Abemaciclib, Palbociclib, Ribociclib	Phase III, multicenter, randomized, open label	ABC, have not previously received systemic therapy. Exclusion: Have received (neo)adjuvant ET or CDK4/6i and had recurrence during or within 12 months of therapy	NCT06760637 [78]
ONO-4578 and Letrozole Plus CDK4 /6 Inhibitors in Breast Cancer	Arm A: ONO-4578 + letrozole + Palbociclib Arm B: ONO-4578 + letrozole + Abemaciclib	Abemaciclib; Palbociclib	Phase I, multicenter, open label	ABC, ECOG 0 to 1	NCT06570031 [79]
Discontinuation of CDK4/6 Inhibitors in Patients with Metastatic HR Positive, HER2 Negative Breast Cancer	Arm A: Continuation of CDK4/6i and ET Arm B: Discontinuation of CDK4/6i, continuation of ET	Abemaciclib; Palbociclib	Phase II, randomized, non-comparative, open-label, multicenter	CDK4/6i + ET for at least 12 months with disease control	NCT06307249 [80]
Gedatolisib as First-Line Treatment for Patients With HR-Positive, HER2-Negative Advanced Breast Cancer (VIKTORIA-2)	Arm A: Gedatolisib + Palbociclib or Ribociclib + Fulvestrant Arm B: Palbociclib or Ribociclib + Fulvestrant	Palbociclib; Ribociclib	Phase III, randomized, open label	ABC which progressed during or within 12 months of adjuvant treatment with ET and have not received prior systemic therapy	NCT06757634 [81]
The CDK4/6 Inhibitor Dosing Knowledge (CDK) Study	Arm A: Indicated Dose of CDK4/6i (dosing regimen per FDA approved drug label) + ET Arm B: Titrated Dose of CDK4/6i (escalation to indicated dose based on tolerability) + ET	Palbociclib; Ribociclib	Phase III, multicenter, randomized, open label	ABC, age 65 years and older	NCT06377852 [82]
Ribociclib vs. Palbociclib in Patients with Advanced Breast Cancer Within the HER2-Enriched Intrinsic Subtype (HARMONIA)	Arm A: Ribociclib + Fulvestrant or Letrozole Arm B: Palbociclib + Fulvestrant or Letrozole Arm C: Paclitaxel +/− Tislelizumab	Palbociclib; Ribociclib	Phase III, multicenter, randomized, open label	ABC, HR+/HER2-, HER2-E or basal-like subtype as per central PAM50 analysis. No prior treatment with CDK4/6i	NCT05207709 [83]
Combination Followed by Maintenance Chemotherapy Versus CDK4/6 Inhibitor Combined with Endocrine Therapy for HR Low/HER2-negative Advanced Breast Cancer	Arm A: nab-paclitaxel, Capecitabine, Vinorelbine Arm B: CDK4/6i + ET	Dalpiciclib; Palbociclib	Phase II, randomized, open label	ABC, HR Low/HER2-negative. HR low defined as 1–50% ER expression by immunohistochemistry (IHC); or ER < 1% and PR ≥ 1%	NCT06176534 [84]
Endocrine Therapy with or Without Abemaciclib (LY2835219) Following Surgery in Participants with Breast Cancer (monarchE)	Arm A: Albemaciclib + ET Arm B: ET	Abemaciclib	Phase III, randomized, open label	Tumor size 5 cm or more, 4 or more positive axillary lymph nodes, no metastasis (M0). Undergone definitive surgery for primary breast cancer	NCT03155997 [58,59]
A Neoadjuvant Study of Abemaciclib (LY2835219) in Postmenopausal Women with Hormone Receptor Positive, HER2 Negative Breast Cancer (neoMONARCH)	Arm A: Abemaciclib + Anastrozole Arm B: Abemaciclib Arm C: Anastrozole	Abemaciclib	Phase II, randomized, open label	Early-stage breast cancer, prior ET is allowed. Exclusion: ABC	NCT02441946 [68]
A Study of Abemaciclib (LY2835219) In Participants with Previously Treated Breast Cancer That Has Spread (MONARCH 1)	Abemaciclib	Abemaciclib	Phase II, single arm, open label	ABC, progression on prior ET	NCT02102490 [48]
A Study of Abemaciclib (LY2835219) Combined with Fulvestrant in Women with Hormone Receptor Positive HER2 Negative Breast Cancer (MONARCH 2)	Arm A: Abemaciclib + Fulvestrant Arm B: Placebo + Fulvestrant	Abemaciclib	Phase III, multicenter, randomized, double blind	ABC, have received (neo)adjuvant ET with progression/metastasis on prior ET ≤ 12 months from the end of adjuvant ET or while receiving first-line ET for metastatic disease	NCT02107703 [49]
A Study of Nonsteroidal Aromatase Inhibitors Plus Abemaciclib (LY2835219) in Postmenopausal Women with Breast Cancer (MONARCH 3)	Arm A: Abemaciclib + Anastrozole or Letrozole Arm B: Placebo + Anastrozole or Letrozole	Abemaciclib	Phase III, multicenter, randomized, double blind	ABC. Exclusion: received (neo)adjuvant ET ≤ 12 months	NCT02246621 [50]
Lasofoxifene Combined with Abemaciclib Compared with Fulvestrant Combined with Abemaciclib in Locally Advanced or Metastatic ER+/HER2− Breast Cancer with an ESR1 Mutation (ELAINEIII)	Arm A: Lasofoxifene + Abemaciclib Arm B: Fulvestrant + Abemaciclib	Abemaciclib	Phase III, multicenter, randomized, parallel-group open label	ABC, no evidence of progression for at least 6 months on an AI/CDK4/6i combination, one or more ESR1 point mutations	NCT05696626 [85]
Chidamide in Combination with Abemaciclib and Endocrinotherapy (Doctor’s Choice) in Breast Cancer Patients Previously Treated with Palbociclib (CINDERELLA)	Chidamide + Abemaciclib + ET	Abemaciclib	Phase Ib/II, single group assignment, open label	ABC, disease recurrence and/or metastasis during or after palbociclib-based regimen in (neo)adjutant setting, no more than 3 lines of prior ET. Exclusion: previously treated by a CDK4/6i other than Palbociclib	NCT05464173 [86]
A Study of Abemaciclib and Radiation Therapy in People with Metastatic Breast Cancer	Maximum tolerated dose of Abemaciclib with palliative radiation therapy	Abemaciclib	Phase I/Ib, multicenter, single arm, open label	Metastatic breast cancer; any prior treatments (ET and chemotherapy), including CDK4/6i are permitted	NCT06678269 [87]
Precision Treatment of HR+ HER2− Advanced Breast Cancer Based on SNF Molecular Subtyping (abemaSNF1/3)	SNF 1 subgroup: Arm A: Fulvestrant + Abemaciclib Arm B: Everolimus + Fulvestrant + Abemaciclib SNF 3 subgroup: Arm A: Fulvestrant + Abemaciclib Arm B: Fluzoparib + Fulvestrant + Abemaciclib	Abemaciclib	Phase Ib/II, randomized, controlled, open label	ABC, with progression after prior CDK4/6i. Two main treatment groups are divided by Similarity Network Fusion (SNF): SNF1/SNF3 subtype	NCT06561022 [88]
Chemotherapy Omission in ER+/HER2− Breast Cancer With 1–3 Positive Lymph Nodes Receiving Extended Adjuvant Abemaciclib (Rainbow)	Arm A: ET + Abemaciclib Arm B: Physician’s choice of chemotherapy + ET	Abemaciclib	Phase III, multicenter, randomized, open label	Localized invasive cancer, 1–3 positive lymph nodes, T1–T2. Exclusion: any prior neoadjuvant therapy, ABC, bilateral breast cancer	NCT06341621 [89]
Study Evaluating Abemaciclib + Aromatase Inhibitors in HR+, HER2− Advanced Breast Cancer Patients (HERMIONE-7)	Abemaciclib + Letrozole/Anastrozole	Abemaciclib	Phase II, multicenter, single arm, open label	ABC	NCT04227327 [90]
Impact of Endocrine Therapy and Abemaciclib on Host and Tumor Immune Cell Repertoire/Function in Advanced ER+/HER2− Breast Cancer	Arm A: Abemaciclib + Fulvestrant Arm B: Abemaciclib + AI (Letrozole/Anastrozole)	Abemaciclib	Phase II, non-randomized, open label	ABC	NCT04352777 [91]
Neoadjuvant Study Chemotherapy vs. Letrozole + Abemaciclib in HR+/HER2− High/Intermediate Risk Breast Cancer Patients (CARABELA).	Arm A: Doxorubicin + Cyclophosphamide + Docetaxel/Paclitaxel Arm B: Letrozole + Abemaciclib	Abemaciclib	Phase II, multicenter, randomized, open label	Stage T2 (>2 cm)–T3, T4b, N0–N2, M0 (stages IIA, IIB, IIIA or IIIB). Ki67 index value (≥20%)	NCT04293393 [92]
Endocrine Therapy with Abemaciclib or Chemotherapy as Initial Metastatic Treatment in ER+/HER2− Breast Cancer (AMBRE)	Arm A: Paclitaxel or Capecitabine Arm B: Abemaciclib + ET	Abemaciclib	Phase III, multicenter, randomized, open label	Metastatic breast cancer with liver/lung/pleural/peritoneal metastases. Exclusion: bone metastases only	NCT04158362 [93]
Multigene Risk Score Combined with Ki-67 Dynamic Assessment in Stratified Neoadjuvant Endocrine Therapy Treatment with or Without CDK4/6 Inhibitors in HR+/HER2− Breast Cancer	Arm A: High risk and insensitive to single AI: Dalpiciclib + Letrozole Arm B: High risk and sensitive to AI/Low risk and insensitive to single agent AI: Letrozole Arm C: High risk and sensitive to AI/Low risk and insensitive to single agent AI: Dalpiciclib + Letrozole Arm D: Low risk and sensitive to single agent AI: Letrozole	Dalpiciclib	Phase II, single center, randomized, open label	Early stage, stratified by multigene testing scoring tool (EndoPredict) as low risk or high risk of recurrence and sensitivity to single-agent AI	NCT06650748 [94]
An Exploratory Clinical Study of CDK4/6i Dalpiciclib Combined with AI Neoadjuvant Therapy for Stage II–III HR+/HER2− Breast Cancer	Dalpiciclib + Letrozole	Dalpiciclib	Phase III/IV single arm, open label	Stage II–III. Exclusion: any prior cancer therapy, ABC, bilateral breast cancer	NCT06613373 [95]
PARP Inhibitor with CDK4/6 Inhibitor and Endocrine Therapy in HR+/HER2− Advanced Breast Cancer	Arm A: Fluzoparib + Dalpiciclib + Fulvestrant Arm B: Dalpiciclib + Fulvestrant	Dalpiciclib	Phase III, multicenter, randomized, open label	ABC, SNF3 subtype	NCT06612814 [96]
An Exploratory Clinical Study of Dalpiciclib and Letrozole Combined with Anlotinib Neoadjuvant Therapy in Stage II–III Postmenopausal HR+/HER2− Early Breast Cancer	Dalpiciclib + Letrozole + Anlotinib	Dalpiciclib	Phase Ia/Ib, single arm, non-randomized	Stage II–III breast cancer. Exclusion: any prior cancer therapy, ABC, bilateral breast cancer	NCT06605690 [97]
Adebrelimab Combined with Dalpiciclib and Standard Endocrine Therapy for HR + /HER2− Breast Cancer	Adebrelimab + Dalpiciclib + ET	Dalpiciclib	Phase II, single arm, open label	Stage II–III breast cancer. Exclusion: prior treatment with CDK4/6i or PD1/PD-L1.	NCT06599216 [98]
Exploration of Dalpiciclib + Tucidinostat in HR+/HER2− Advanced Breast Cancer After Failure of CDK4/6 Inhibitor	Dalpiciclib + Tucidinostat + ET	Dalpiciclib	Phase II, single arm, open label	ABC, prior treatment with CDK4/6i	NCT06556862 [99]
Efficacy and Safety of Dalpiciclib Plus Toremifene in the Treatment of Advanced First-line HR Positive and HER2 Negative Breast Cancer: A Multicenter, Single Arm, Exploratory Phase II Clinical Study	Dalpiciclib + Toremifen	Dalpiciclib	Phase II, multicenter, single arm	ABC. Exclusion: any prior treatment with CDKi, Tamoxifen, Everolimus, PI3K inhibitors	NCT06495515 [100]
Apatinib Combined With cdk4/6i in First-line Treatment for HR+/HER2− SNF4 Subtype Breast Cancer	Arm A: Apatinib + Dalpiciclib + ET Arm B: Dalpiciclib + ET	Dalpiciclib	Phase III, randomized, open label	ABC, no prior CDK4/6i use or > one year since last dose of CDK4/6i	NCT06447623 [101]
Efficacy and Safety of Dalpiciclib with Endocrine Therapy as Adjuvant Treatment in HR+/HER2− Early Breast Cancer	Dalpiciclib + ET (Fulvestrant/AI)	Dalpiciclib	Phase II, multicenter, single arm, open label	Stage IIA-IIIC stage (T2-4N0-3M0) stage IIA only includes T1N1M0. Exclusion: metastatic breast cancer	NCT06341894 [102]
A Study of SHR6390 in Combination with Fulvestrant in Patients with HR Positive and HER2 Negative Advanced Breast Cancer (DAWNA-1)	Arm A: Dalpiciclib + Fulvestrant Arm B: Placebo + Fulvestrant	Dalpiciclib	Phase III, multicenter, randomized, double blind	ABC. One previous line of chemotherapy is allowed, in addition to ET	NCT03927456 [55]
A Study of SHR6390 in Combination with Letrozole or Anastrozole in Patients with HR Positive and HER2 Negative Advanced Breast Cancer (DAWNA-2)	Arm A: Dalpiciclib + Letrozole/Anastrozole Arm B: Placebo + Letrozole/Anastrozole	Dalpiciclib	Phase III, multicenter, randomized, double blind	ABC	NCT03966898 [56]
Locoregional Treatment and Palbociclib in de Novo, Treatment Naive, Stage IV ER+, HER2− Breast Cancer Patients (PALATINE)	Palbociclib + Locoregional treatment: Surgery, surgery + radiotherapy or radiotherapy	Palbociclib	Phase II, multicenter, single arm, open label	Any T or N stage, with metastasis. Exclusion: advanced, symptomatic, visceral spread at risk of life-threatening complications	NCT03870919 [103]
Neoadjuvant Response-guided Treatment of Slowly Proliferating Hormone Receptor Positive Tumors (PREDIX LumA)	Arm A: ET Arm B: ET + Palbociclib Arm C: ET + Palbociclib	Palbociclib	Phase II, randomized, open label	Exclusion: Metastatic breast cancer. Patients receive ET, with their response noted at 4 weeks. If responsive (defined by ≥20% decrease in Ki67), they are randomized to Arm A or B. If stable (<20% decrease or increase in Ki67 without radiological progression), they are randomized to Arm C	NCT02592083 [104]
Neoadjuvant Response-guided Treatment of Luminal B-type Tumors and Luminal A-type Tumors with Node Metastases (PREDIX LumB)	Arm A: Paclitaxel Arm B1: Tamoxifen + Palbociclib Arm B2: AI + Palbociclib Arm B3: AI + Goserelin + Palbociclib	Palbociclib	Phase II, randomized, open label	Luminal B type. Exclusion: metastasis	NCT02603679 [105]
Trial of Anastrozole and Palbociclib in Metastatic HER2-Negative Breast Cancer	Arm A: Anastrozole + Palbociclib Arm B: Maintenance therapy Anastrozole + Palbociclib	Palbociclib	Phase II, open label	ABC. Arm A: previous (neo)adjuvant ET is allowed Arm B: only first-line chemotherapy in metastatic disease with response	NCT03425838 [106]
PAlbociclib Plus Tamoxifen for the Treatment of Hormone Receptor-positive, HER2-negative Advanced Breast Cancer Women—Asian study (PATHWAY)	Arm A: Palbociclib + Tamoxifen ± Goserelin Arm B: Placebo + Tamoxifen ± Goserelin	Palbociclib	Phase III, randomized, quadruple blind	ABC. Exclusion: prior treatment with any CDKi, tamoxifen, everolimus, or agent that inhibits the PI3K-mTOR pathway	NCT03423199 [107]
Palbociclib With Fulvestrant for Metastatic Breast Cancer After Treatment with Palbociclib and an Aromatase Inhibitor	Palbociclib + Fulvestrant	Palbociclib	Phase II, single arm, open label	ABC. Prior therapy on Palbociclib and AI. No prior therapy with fulvestrant, everolimus or PI3K-mTOR inhibitors	NCT02738866 [108]
Endocrine Therapy Fulvestrant & Palbociclib or Aromatase Inhibitor Therapy in Treating Older Patients with Hormone Responsive Breast Cancer That Cannot Be Removed by Surgery	Palbociclib + Fulvestrant	Palbociclib	Phase II, single arm, open label	ABC or patient refusing breast surgery, vulnerable or frail by Balducci Criteria	NCT02760030 [109]
Palbociclib and Endocrine Therapy for LObular Breast Cancer Preoperative Study (PELOPS)	Arm A: Tamoxifen followed by ET Arm B: Letrozole followed by ET Arm C: Tamoxifen followed by ET + Palbociclib Arm D: Letrozole followed by ET + Palbociclib	Palbociclib	Phase II, multicenter, randomized, open label	Stage I–III breast cancer. Exclusion: Stage IV	NCT02764541 [110]
Efficacy and Safety of Giredestrant Combined with Palbociclib Compared with Letrozole Combined with Palbociclib in Participants with Estrogen Receptor-Positive, HER2-Negative Locally Advanced or Metastatic Breast Cancer (persevERA Breast Cancer)	Arm A: Giredestrant + Letrozole-matched Placebo + Palbociclib Arm B: Letrozole + Giredestrant-matched Placebo + Palbociclib	Palbociclib	Phase III, multicenter, randomized, double blind	ABC Exclusion: prior treatment with Selective Estrogen Receptor Degrader (SERD)	NCT04546009 [111]
Phase 1/2 Study of Amcenestrant (SAR439859) Single Agent and in Combination with Other Anti-cancer Therapies in Postmenopausal Women with Estrogen Receptor Positive Advanced Breast Cancer (AMEERA-1)	Each Arm has an initial dose escalation followed by a dose expansion part. Arm A: Amcenestrant Arm B: Amcenestrant + Palbociclib Arm C: Amcenestrant + Alpelisib Arm D: Amcenestrant + Everolimus	Palbociclib	Phase I/II, multicenter, randomized, open label	ABC, prior treatment with ET within the last 6 months	NCT03284957 [112]
Palbociclib for HR Positive/HER2-negative Isolated Locoregional Recurrence of Breast Cancer (POLAR)	Arm A: Palbociclib + ET Arm B: ET only	Palbociclib	Phase III, multicenter, randomized, open label	Ipsilateral local/regional recurrence in breast, chest wall, axillary lymph nodes. Surgery within 6 months or radiotherapy within 2 weeks prior to study. Exclusion: recurrence not surgically removable, distant metastasis	NCT03820830 [113]
Palbociclib and Circulating Tumor DNA for ESR1 Mutation Detection (PADA-1)	Arm A: Palbociclib + AI (Anastrozole, Letrozole, Exemestane) Arm B: Palbociclib + Fulvestrant	Palbociclib	Phase III, multicenter, randomized, open label	ABC, ESR1 screening, no metastasis on endocrine therapy. All patients to initially receive Palbociclib + AI. Patients with progression or increase in ESR1 circulating level then randomized to Arm A or Arm B with plan for cross over	NCT03079011 [114]
Study Of Letrozole with Or Without Palbociclib (PD-0332991) For the First-Line Treatment of Hormone-Receptor Positive Advanced Breast Cancer (PALOMA-1/TRIO-18)	Arm A: Letrozole + Palbociclib Arm B: Letrozole	Palbociclib	Phase II, multicenter, randomized, open label	ABC	NCT00721409 [41]
Palbociclib (PD-0332991) + Letrozole versus Letrozole For 1st Line Treatment of Postmenopausal Women with ER + /HER2− Advanced Breast Cancer (PALOMA-2)	Arm A: Letrozole + Palbociclib Arm B: Letrozole	Palbociclib	Phase III, multicenter, randomized, open label	ABC. Exclusion: metastasis/progression within 12 months of prior ET.	NCT01740427 [42]
Palbociclib (PD-0332991) Combined with Fulvestrant in Hormone Receptor+ HER2-Negative Metastatic Breast Cancer After Endocrine Failure (PALOMA-3)	Arm A: Fulvestrant + Palbociclib Arm B: Placebo + Fulvestrant	Palbociclib	Phase III, multicenter, randomized, triple blind	ABC, history of progression/metastasis on ET. Exclusion: no prior therapy with CDK4/6i, Fulvestrant, Everolimus, PI3K or mTOR inhibitor	NCT01942135 [43]
Palbociclib (PD-0332991) + Letrozole VS. Placebo+ Letrozole For 1st Line Treatment of Asian Postmenopausal Women With ER+/HER2− Advanced Breast Cancer (PALOMA-4)	Arm A: Fulvestrant + Palbociclib Arm B: Placebo + Fulvestrant	Palbociclib	Phase III, multicenter, randomized, quadruple blind	ABC, Asian postmenopausal female	NCT02297438 [115]
Palbociclib CoLlaborative Adjuvant Study (PALLAS)	Arm A: Palbociclib + ET Arm B: ET	Palbociclib	Phase III, multicenter randomized, open label	Stage II to early Stage III, undergone definitive breast surgery for current malignancy	NCT02513394 [62]
Palbociclib in Addition to Standard Endocrine Treatment in Hormone Receptor Positive Her2 Normal Patients with Residual Disease After Neoadjuvant Chemotherapy and Surgery (PENELOPE-B)	Arm A: Palbociclib Arm B: Placebo	Palbociclib	Phase III, multicenter, randomized, quadruple blind	Early stage, with adequate surgical resection—histologically complete resection R0 in breast conservative surgery or R1 after total mastectomy	NCT01864746 [63]
Efficacy of Letrozole + Palbociclib Combination as Neoadjuvant Treatment of Stage II–IIIA PAM 50 ROR-defined Low or Intermediate Risk Luminal Breast Cancer, in Postmenopausal Women (NeoPAL)	Arm A: Fluorouracile+ Epirubicine+ Cyclophosphamide+ Docetaxel then Docetaxel only Arm B: Letrozole + Palbociclib	Palbociclib	Phase II, randomized, open label	Stage II to IIIA, M0, luminal A + proven nodal involvement or luminal B through PAM50	NCT02400567 [64]
Evaluating the Biological and Clinical Effects of the Combination of Palbociclib with Letrozole as Neoadjuvant Therapy in Post-Menopausal Women with Estrogen-Receptor Positive Primary Breast Cancer (PALLET)	Arm A: Letrozole Arm B: Letrozole then Letrozole + Palbociclib Arm C: Palbociclib then Letrozole + Palbociclib Arm D: Letrozole + Palbociclib	Palbociclib	Phase II, randomized, open label	Early stage, operable ER-positive/HER2-negative, invasive early breast cancer	NCT02296801 [66]
PD 0332991 and Anastrozole for Stage 2 or 3 Estrogen Receptor Positive and HER2 Negative Breast Cancer (NeoPalAna)	Arm A: PIK3CA Wild Type Cohort Palbociclib + Letrozole Arm B: PIK3CA Mutant Type Cohort Palbociclib + Letrozole Arm C: Endocrine Resistant Cohort Palbociclib + Letrozole	Palbociclib	Phase II, single arm	Clinical T2-T4c, any N, M0. No prior history with CDK4/6i	NCT01723774 [116]
Neoadjuvant Letrozole and Palbociclib in Patients with Stage II–IIIB Breast Cancer, HR+/HER2− (DxCARTES)	Letrozole + Palbociclib	Palbociclib	Phase II, multicenter, open-label	Early stage: T2–4, N0–2, M0	NCT03819010 [117]
Safety, Tolerability, and Preliminary Efficacy of Sirolimus (Albumin-bound) in Combination with Palbociclib and Fulvestrant in Patients with Advanced HR-Positive, HER2-Negative Breast Cancer	Sirolimus: Dose escalation, intravenous infusion; Palbociclib and Fulvestrant	Palbociclib	Phase II; single group, open label	Previously received (neo)adjuvant endocrine therapy (ET)	NCT06856200 [118]
Efficacy and Safety of Inavolisib Plus CDK4/6 Inhibitor and Letrozole vs. Placebo + CDK4/6i and Letrozole in Participants with Endocrine-Sensitive PIK3CA-Mutated, Hormone Receptor-Positive, HER2-Negative Advanced Breast Cancer (INAVO123)	Arm A: Inavolisib + Letrozole + CDK4/6i Arm B: Placebo + Letrozole + CDK4/6i	Palbociclib	Phase III, multicenter, randomized, double blind, placebo-controlled	PIK3CA mutated advanced breast cancer (ABC), de novo or relapsed after at least 2 years of (neo)adjuvant ET without disease progression or disease-free interval of 1 year	NCT06790693 [119]
Vepdegestrant (ARV-471, PF-07850327) Plus Palbociclib Versus Letrozole Plus Palbociclib in Participants with Estrogen Receptor Positive, Human Epidermal Growth Factor Negative Advanced Breast Cancer (VERITAC-3)	Arm A: Vepdegestrant + Palbociclib Arm B: Letrozole + Palbociclib	Palbocicblib	Phase III, multicenter, randomized, open label	ABC, no prior systemic treatment including ET	NCT05909397 [120]
Gedatolisib Plus Fulvestrant with or Without Palbociclib vs. Standard-of-Care for the Treatment of Patients with Advanced or Metastatic HR+/HER2− Breast Cancer (VIKTORIA-1)	Arm A: Gedatolisib + Palbociclib + Fulvestrant Arm B: Gedatolisib + Fulvestrant Arm C: Fulvestrant Arm D: Gedatolisib + Palbociclib + Fulvestrant Arm E: Alpelisib + Fulvestrant	Palbociclib	Phase III, multicenter, randomized, open label	ABC, progressed during or after CDK4/6 inhibitor combination treatment with AI. Arm A, B and C included patients without PIK3CA Mutations (wild type) Arm D and E included patients with PIK3CA mutation (mutated type) Exclusion: prior treatment with a PI3K inhibitor, A protein kinase B (Akt) inhibitor, or mTOR inhibitor	NCT05501886 [121]
Prediction of Treatment Efficacy of the Combination of Palbociclib/(Letrozole or Anastrozole) in First Line Metastatic Women with Luminal, HER2 Negative Advanced Breast Cancer, Using Infrared Laser Spectroscopy Analysis on Liquid Biopsies. (ICRG0201)	Palbociclib + Letrozole or Anastrozole	Palbociclib	Phase II, single treatment arm multicenter, open label	Clinical stage IIIb/IV. Exclusion: patients with bilateral breast cancer. The aim of the study is to assess predictive capability of infrared laser spectroscopy analysis on liquid biopsies in terms of treatment efficacy	NCT05190094 [122]
Clinical Trial Assessing the Safety of Neoadjuvant Palbociclib in Combination with Endocrine Therapy	Palbociclib + Letrozole	Palbociclib	Phase II, single treatment group, open label	Early-stage cancer (2 cm or greater or <2 cm in size with lymph node involvement)	NCT05069038 [122]
Study to Evaluate the Extended Overall Survival (OS) Data from PARSIFAL Study (The PARSIFAL-LONG Study)	Arm A: Fulvestrant + Palbociclib Arm B: Letrozole + Palbociclib	Palbociclib	Phase III, multicenter, randomized, open label	ABC, have not previously received systemic therapy	NCT06525675 [123]
Palbociclib After CDK and Endocrine Therapy (PACE)	Arm A: Fulvestrant Arm B: Fulvestrant + Palbociclib Arm C: Fulvestrant + Palbociclib + Avelumab	Palbociclib	Phase II, multicenter, randomized, open label	ABC, with progression on CDK4/6i and ET	NCT03147287 [124]
Pembrolizumab, Endocrine Therapy, and Palbociclib in Treating Postmenopausal Patients with Newly Diagnosed Metastatic Stage IV Estrogen Receptor Positive Breast Cancer	Arm A: Letrozole/Fulvestrant + Pembrolizumab + Palbociclib Arm B: Letrozole + Palbociclib + pembrolizumab	Palbociclib	Phase II, open label	Metastatic Stage IV breast cancer, no prior use of Pembrolizumab or PD-L1 inhibitors	NCT02778685 [125]
Efficacy and Safety of LEE011 in Postmenopausal Women with Advanced Breast Cancer. (MONALEESA-2)	Arm A: Ribociclib + Letrozole Arm B: Placebo + Letrozole	Ribociclib	Phase III, multicenter, randomized, quadruple blind	ABC. Exclusion: prior CDK4/6i therapy	NCT01958021 [45]
Ribociclib and Fulvestrant in Hormone Receptor-Positive, Human Epidermal Growth Factor Receptor 2-Negative Advanced Breast Cancer: MONALEESA-3	Arm A: Ribociclib + Fulvestrant Arm B: Placebo + Fulvestrant	Ribociclib	Phase III, multicenter, randomized, double blind	ABC. Further stratified based on prior ET, presence of liver and/or lung metastasis	NCT02422615 [46]
Efficacy and Safety in Premenopausal Women with Hormone Receptor Positive, HER2-negative Advanced Breast Cancer (MONALEESA-7)	Arm A: Ribociclib + NSAI/tamoxifen + goserelin Arm B: Placebo + NSAI/tamoxifen + goserelin	Ribociclib	Phase III, multicenter, randomized, quadruple blind	ABC, had received no (neo)adjuvant therapy	NCT02278120 [47]
Fulvestrant With Ribociclib Versus Physician’s Choice Treatments Recurred After Completion of Adjuvant Cyclin-Dependent Kinase 4/6 Inhibitors in HR+, HER2− Metastatic Breast Cancer	Arm A: Ribociclib + fulvestrant Arm B: Fulvestrant or Exemestane + Everolimus	Ribociclib	Phase III, randomized, open label	ABC, patient must have received either at least one year of adjuvant abemaciclib or ribociclib and recurrence of cancer should be greater than one year from last dose of adjuvant CDK4/6i	NCT06849947 [126]
Efficacy and Safety of Ribociclib Combined with AI Versus Physician’s Choice of Chemotherapy Sequential Endocrine Therapy in ER Middle-low-expression/HER2-negative Advanced Breast Cancer (Rachel)	Arm A: Chemotherapy + Sequential Ribociclib + AI Arm B: Ribociclib + AI	Ribociclib	Phase II, randomized, open label	ABC, patients who have not received systematic chemotherapy or (neo)adjuvant CDK4/6i previously	NCT06656624 [127]
Efficacy and Safety of Ribociclib in Combination with NSAI Versus Physician’s Choice of Chemotherapy Sequential Endocrine Therapy in HR+/HER2− Advanced Breast Cancer	Arm A: Chemotherapy + Sequential Ribociclib + Anastrozole or Letrozole Arm B: Ribociclib + Anastrozole or Letrozole	Ribociclib	Phase II, randomized, open label	ABC, patient has not received systemic anticancer therapy at stage of recurrence/metastasis	NCT06375707 [128]
Neoadjuvant Multi-agent Chemotherapy or Letrozole Plus Ribociclib in Luminal B/HER2-negative Breast Cancer. (CORALLEEN)	Arm A: Ribociclib + Letrozole Arm B: Doxorubicin + Cyclophosphamide + Paclitaxel	Ribociclib	Phase II, randomized, open label	Stage I to Stage IIIA, M0, Luminal Subtype B by PAM50 analysis	NCT02712723 [65]
A Trial to Evaluate Efficacy and Safety of Ribociclib with Endocrine Therapy as Adjuvant Treatment in Patients With HR+/HER2− Early Breast Cancer (NATALEE)	Arm A: Ribociclib + ET Arm B: ET	Ribociclib	Phase III, randomized, open label	Stage II–III, with tumor free microscopic margins post-surgical resection, completed adjuvant radiotherapy as indicated	NCT03701334 [60]
Efficacy and Safety of Ribociclib in Pre- and Postmenopausal Chinese Women with HR Positive, HER2-negative, Advanced Breast Cancer.	Arm A: Premenopausal: NSAI + Goserelin + Ribociclib NSAI: Anastrozole/Letrozole Postmenopausal: Letrozole + Ribociclib Arm B: Premenopausal: NSAI + Goserelin + Placebo Postmenopausal: Letrozole + Placebo	Ribociclib	Phase II, multicenter, randomized, quadruple blind	ABC	NCT03671330 [129]
FACILE: FeAsibility of First-line RiboCIclib in OLdEr Patients with Advanced Breast Cancer (FACILE)	Ribociclib + Anastrozole/Letrozole + Triptorelin/Leuprolide/Goserelin	Ribociclib	Phase II, multicenter, single arm	ABC. Age 70 or older	NCT03944434 [130]
Letrozole Plus Ribociclib or Placebo as Neo-adjuvant Therapy in ER-positive, HER2-negative Early Breast Cancer (FELINE)	Arm A: Placebo + Letrozole Arm B: Ribociclib (21 days on/7 days off) + Letrozole Arm C: Ribociclib (Continuous daily dosing) + Letrozole	Ribociclib	Phase II, multicenter, randomized, quadruple blind	Early breast cancer/clinical Stage II–III.	NCT02712723 [67]
Neoadjuvant Treatment of Locally advanced Breast Cancer Patients with Ribociclib and Letrozole (NEOLETRIB)	Ribociclib + Letrozole	Ribociclib	Phase II, multicenter, single arm	Locally advanced breast cancer, T2 (>3 cm in diameter) or T3–T4, and/or N2–3. Exclusion: metastatic breast cancer	NCT05163106 [131]

**Table 2:** Clinical trials involving CDK4/6i in HER2-positive breast cancer.

Study Name	Intervention	CDK-Inhibitor	Phase	Patient Population	NCT
Palbociclib Combine with Anti-HER2 Therapy in Triple Positive Advanced Breast Cancer	Palbociclib + Trastuzumab + Pertuzumab Letrozole/Exemestane	Palbociclib	Phase II, single arm, multicenter, open label	ABC, HR+/HER2+	NCT05969184 [153]
Palbociclib, Trastuzumab, Pyrotinib and Fulvestrant Treatment in Patients with Brain Metastasis From ER/PR Positive, HER-2 Positive Breast Cancer: A Multi-center, Prospective Study in China	Palbociclib + Trastuzumab + Pyrotinib + Fulvestrant	Palbociclib	Phase II, multicenter, single arm, open label	metastatic BC with brain metastasis. Exclusion: uncontrolled cNS symptoms due to metastasis, increasing doses of steroids, leptomeningeal disease or history of whole brain radiotherapy	NCT04334330 [151]
Neoadjuvant Treatment with Palbociclib and Exemestane Plus Trastuzumab and Pyrotinib in Estrogen Receptor (ER)-Positive, HER2-positive Breast Cancer (neoPEHP)	Palbociclib + Exemestane + Trastuzumab + Pyrotinib	Palbociclib	Phase II, single arm, open label	Stage cT1c and cT4a–d. Exclusion: metastatic breast cancer or bilateral breast cancer	NCT04858516 [164]
To Reduce the Use of Chemotherapy in Postmenopausal Patients With ER-positive and HER2-positive Breast Cancer (TOUCH)	Arm A: Paclitaxel + Trastuzumab + Pertuzumab Arm B: Palbociclib + Paclitaxel + Trastuzumab + Pertuzumab	Palbociclib	Phase II, multicenter, randomized, open label	Early-stage breast cancer, >1 cm tumor size, cN0-cN1	NCT03644186 [161,162,163]
T-DM1 and Palbociclib for Metastatic HER2 Breast Cancer (T-DM1)	Arm A: Palbociclib + ado-trastuzumab emtansine (T-DM1) Arm B: T-DM1	Palbociclib	Phase II, multicenter, randomized	ABC	NCT03530696 [145]
A Multicenter, Phase I/II Trial of Anastrozole, Palbociclib, Trastuzumab and Pertuzumab in HR-positive, HER2-positive Metastatic Breast Cancer	Anastrozole + Palbociclib + Trastuzumab + Pertuzumab	Palbociclib	Phase Ib/II, multicenter, single arm	Metastatic breast cancer. Stable brain metastasis allowed	NCT03304080 [155]
Tucatinib, Palbociclib and Letrozole in Metastatic Hormone Receptor Positive and HER2-positive Breast Cancer	Tucatinib + Palbociclib + Letrozole	Palbociclib	Phase Ib/II, multicenter, single arm, open label	ABC, stable brain metastasis allowed. Prior treatment with at least two HER2-targeted agents (trastuzumab, pertuzumab, TDM-1)	NCT03054363 [146]
Neo-Adjuvant Treatment with Palbociclib: Effect on Ki67 and Apoptosis Before, During and After Treatment (NA-PHER2)	Trastuzumab, Pertuzumab, Palbociclib ± Fulvestrant	Palbociclib	Phase II, multicenter, open label	Early-stage breast cancer, HR+HER2+ and HR+HER2-low	NCT02530424 [158,159,160]
Palbociclib and Trastuzumab with Endocrine Therapy in HER2-positive Metastatic Breast Cancer (PATRICIA II)	Arm A: HR-/HER2+ Palbociclib + Trastuzumab Arm B1: HR+/HER2+ Palbociclib + Trastuzumab Arm B2: HR+/HER2+ Palbociclib + Trastuzumab + Letrozole Arm C1: HR+/HER2+ Luminal subtype Palbociclib + Trastuzumab+ ET Arm C2: HR+/HER2+ Luminal subtype TDM-1/chemotherapy/ET + Trastuzumab	Palbociclib	Phase II, multicenter, open label, Simon’s two-stage-design study	ABC, with cohorts A, B1 and B2 divided based on HR±/HER2+ and cohort C1 and C2 based on HR+HER2+ and luminal intrinsic subtype based on PAM50	NCT02448420 [149,150]
Randomized, Open Label, Clinical Study of the Targeted Therapy, Palbociclib, to Treat Metastatic Breast Cancer (PATINA)	Arm A: Palbociclib + ET + Trastuzumab or Pertuzumab Arm B: ET + Trastuzumab or Pertuzumab	Palbociclib	Phase III, multicenter, randomized, open label	ABC. If received anti-HER2 therapy is should have been >6 months prior to metastasis. No prior CDK4/6i	NCT02947685 [155,156,157]
Abemaciclib (LY2835219) in Women With HR+/HER2+ Locally Advanced or Metastatic Breast Cancer (monarcHER)	Arm A: Abemaciclib + Trastuzumab +Fulvestrant Arm B: Abemaciclib + Trastuzumab Arm C: Trastuzumab + Systemic Chemotherapy	Abemaciclib	Phase II, multicenter, open label	ABC, received prior anti-HER2 therapy	NCT02675231 [147,148]
Pyrotinib, Dalpiciclib (SHR6390) and Endocrine Therapy in Subjects with Dual-receptor Positive (ER+/HER2+) Advanced Breast Cancer (PLEASURABLE)	Dalpiciclib + Pyrotinib + Letrozole/Fulvestrant	Dalpiciclib	Phase Ib/II, single arm, open label	ABC, HR+/HER2+ recurrent or metastatic breast cancer. Received no more than 1 prior anti-HER2 therapy. Has not received anti-HER TKI	NCT03772353 [152]
Pyrotinib Combined With Trastuzumab, Dalpiciclib and Letrozole for HR+/HER2+ Breast Cancer	Pyrotinib + Trastuzumab + Dalpiciclib + Letrozole	Dalpiciclib	Phase Ib/II, single arm, open label	Early (T2–3, N0–1, M0) or locally advanced (T2–3, N2–3, M0). Exclusion: Metastatic breast cancer	NCT05800756 [165]
Study to Evaluate the Efficacy and Safety of SHR6390 Combined with Pyrotinib in HER2+ Advanced Breast Cancer (DAP-Her-01)	Pyrotinib + Dalpiciclib	Dalpiciclib	Phase Ib/II, single arm, open label	ABC, age 18–70	NCT04293276 [143]
Treatment of Dalpiciclib Combined with Pyrotinib for Trastuzumab-sensitive HER2+ Advanced Breast Cancer (DAP-Her-02)	Arm A: Dalpiciclib + Pyrotinib + Fulvestrant Arm B: Dalpiciclib + Pyrotinib + Inetetamab	Dalpiciclib	Phase II, Randomized, open label	ABC. Group A HR+/HER2+, Group B: HR-/HER2+	NCT05328440 [144]
T-DM1 Combined with CDK4/6 Inhibitor Ribociclib	T-DM1 + Ribociclib	Ribociclib	Phase II, multicenter, single arm, open label	aBc, prior treatment with trastuzumab and taxanes	NCT06481956 [154]

## Supplementary Materials

The following supporting information can be downloaded at: https://www.mdpi.com/article/10.3390/cancers17172788/s1, Table S1: Drug Categories and Mechanisms; Table S2: List of Abbreviations.
