# Supplementary material for: Efficacy and Predictability of Cyclin-Dependent Kinase 4/6 Inhibitors in HER2-Positive Breast Cancer
Source: Cancers (Basel). 2025 Aug 26;17(17):2788. doi: 10.3390/cancers17172788 (PMC12427547; doi:10.3390/cancers17172788)
Supplement: Supplementary file 1 [file cancers-17-02788-s001.zip › Table S1. drug classes.pdf]

Supplemental Table S1. Drug Categories and Mechanisms.

Drug Name	Drug Class	Mechanism of Action
Palbociclib	CDK4/6 Inhibitor	Inhibits CDK4/6 to block phosphorylation of Rb protein, inducing G1 cell cycle arrest.
Ribociclib	CDK4/6 Inhibitor	Selective CDK4/6 inhibitor causing G1 phase arrest by preventing Rb phosphorylation.
Abemaciclib	CDK4/6 Inhibitor	CDK4/6 inhibitor with higher CDK4 selectivity; allows continuous dosing due to lower neutropenia.
Dalpiciclib	CDK4/6 Inhibitor	Investigational selective CDK4/6 inhibitor used in advanced breast cancer trials.
Flavopiridol*	Pan-CDK Inhibitor	First-generation CDK inhibitor, not selective, with significant toxicity.
Roscovitine*	Pan-CDK Inhibitor	Non-selective CDK inhibitor, lacked efficacy in trials.
Dinaciclib*	Multi-CDK Inhibitor	Inhibits CDK1/2/5/9; under investigation for hematologic and solid tumors.
Trastuzumab	HER2 Monoclonal Antibody	Binds HER2 extracellular domain to inhibit dimerization and mediate ADCC.
Pertuzumab	HER2 Monoclonal Antibody	Prevents HER2 dimerization with other HER receptors; used with trastuzumab.

Margetuximab	HER2 Monoclonal Antibody	Fc-engineered HER2-targeted antibody to enhance ADCC response.
Inetetamab*	HER2 Monoclonal Antibody	Trastuzumab biosimilar used in investigational combination regimens.
Lapatinib	HER2 TKI	Dual EGFR/HER2 TKI that inhibits intracellular phosphorylation of HER2.
Neratinib	HER2 TKI	Irreversible pan-HER TKI that blocks EGFR, HER2, and HER4 activity.
Tucatinib	HER2 TKI	Selective HER2 TKI with reduced EGFR inhibition, minimizing off-target effects.
Pyrotinib	HER2 TKI	Irreversible pan-HER TKI under clinical investigation, especially in Asia.
Trastuzumab Emtansine (T-DM1)	HER2 ADC	HER2-targeted antibody linked to a cytotoxic agent (DM1) to deliver chemotherapy directly to cancer cells.
Trastuzumab Deruxtecan	HER2 ADC	HER2-targeted antibody linked to topoisomerase I inhibitor, releasing cytotoxin upon internalization.
Letrozole	Aromatase Inhibitor	Blocks estrogen synthesis by inhibiting aromatase enzyme.
Anastrozole	Aromatase Inhibitor	Inhibits aromatase enzyme to lower estrogen levels.
Exemestane	Aromatase Inhibitor	Steroidal aromatase inhibitor causing irreversible enzyme inactivation.

Fulvestrant	Selective Estrogen Receptor Degrader (SERD)	Binds and degrades ER, inhibiting estrogen signaling.
Giredestrant*	SERD	Oral investigational SERD under evaluation for ER+ breast cancer.
Elacestrant	SERD	Investigational oral SERD effective in ESR1-mutant cancers.
Camizestrant*	SERD	Next-gen oral SERD with potent ER degradation activity.
Capecitabine	Chemotherapy	Prodrug converted to 5-FU; inhibits DNA synthesis.
Vinorelbine	Chemotherapy	Inhibits microtubule assembly to arrest cell division.
Paclitaxel	Chemotherapy	Stabilizes microtubules and prevents their disassembly during mitosis.
Docetaxel	Chemotherapy	Inhibits microtubule depolymerization to induce apoptosis.
Doxorubicin	Chemotherapy	Intercalates DNA and inhibits topoisomerase II.
Cyclophosphamide	Chemotherapy	Alkylating agent causing DNA cross-links and strand breakage.
Alpelisib	PI3K Inhibitor	Inhibits PI3Kα isoform; effective in PIK3CA-mutant breast cancer.
Everolimus	mTOR Inhibitor	Inhibits mTOR pathway, reducing protein synthesis and proliferation.

Gedatolisib*	Dual PI3K/mTOR Inhibitor	Targets both PI3K and mTOR pathways; under clinical evaluation.
Saruparib*	PARP Inhibitor	Blocks PARP-mediated DNA repair in BRCA-mutated cells.
Fluzoparib*	PARP Inhibitor	Oral PARP inhibitor targeting tumor cells with homologous recombination defects.
Tucidinostat*	HDAC Inhibitor	Epigenetic modifier affecting chromatin remodeling and gene expression.
Chidamide*	HDAC Inhibitor	Selective HDAC inhibitor under study for combination regimens.
Apatinib*	VEGFR-2 Inhibitor	Blocks angiogenesis by inhibiting VEGFR-2 signaling.
Adebrelimab*	PD-L1 Inhibitor	Immune checkpoint inhibitor targeting PD-L1.
Vepdegestrant*	Selective ER Degrader (SERD)	Degrades estrogen receptors to block hormone signaling.
Amcenestrant*	SERD	Oral SERD targeting ERα with anti-proliferative activity.
ONO-4578*	Immunotherapy Agent	Investigational immune modulator targeting unknown immune checkpoint pathways.

All drugs denoted by an asterisk (*) are currently under investigation and pending full FDA approval for use in Breast Cancer
