# Supplementary material for: Efficacy and Predictability of Cyclin-Dependent Kinase 4/6 Inhibitors in HER2-Positive Breast Cancer
Source: Cancers (Basel). 2025 Aug 26;17(17):2788. doi: 10.3390/cancers17172788 (PMC12427547; doi:10.3390/cancers17172788)
Supplement: Supplementary file 1 [file cancers-17-02788-s001.zip › Table S2. abbreviations.pdf]

Supplemental Table S2: List of Abbreviations.

Abbreviation	Full Term
ABC	Advanced Breast Cancer
ADC	Antibody-Drug Conjugate
AI	Aromatase Inhibitor
AKT	Protein Kinase B
APC/C	Anaphase-Promoting Complex/Cyclosome
ASCO	American Society of Clinical Oncology
BC	Breast Cancer
CDK	Cyclin Dependent Kinase
CDK4/6i	Cyclin Dependent Kinase 4/6 Inhibitor
CKI	Cyclin Dependent Kinase Inhibitor
CNS	Central Nervous System
DNA	Deoxyribonucleic Acid
EGFR	Epidermal Growth Factor Receptor
ER	Estrogen Receptor
ERE	Estrogen Response Element
ET	Endocrine Therapy
FDA	Food and Drug Administration
HER2	Human Epidermal Growth Factor Receptor 2
HR	Hormone Receptor
iDFS	Invasive Disease-Free Survival
IHC	Immunohistochemistry
MAPK	Mitogen-Activated Protein Kinase

MBC	Metastatic Breast Cancer
mTOR	Mechanistic Target of Rapamycin
NSAI	Non-Steroidal Aromatase Inhibitor
ORR	Overall Response Rate
OS	Overall Survival
PFS	Progression-Free Survival
PI3K	Phosphoinositide 3-Kinase
PR	Progesterone Receptor
Rb	Retinoblastoma Protein
RTK	Receptor Tyrosine Kinase
TKI	Tyrosine Kinase Inhibitor
